# Tamoxifen Increased Parasite Burden and Induced a Series of Histopathological and Immunohistochemical Changes During Chronic Toxoplasmosis in Experimentally Infected Mice

**DOI:** 10.3389/fmicb.2022.902855

**Published:** 2022-05-30

**Authors:** Ashraf Mohamed Barakat, Hassan Ali Mohamed El Fadaly, Rabab Fawzy Selem, Abd El-Nasser A. Madboli, Khaled A. Abd El-Razik, Ehssan Ahmed Hassan, Ali H. Alghamdi, Ehab Kotb Elmahallawy

**Affiliations:** ^1^Department of Zoonotic Diseases, Veterinary Research Institute, National Research Centre, Giza, Egypt; ^2^Department of Parasitology, Faculty of Medicine, Benha University, Benha, Egypt; ^3^Department of Animal Reproduction, Veterinary Research Institute, National Research Centre, Giza, Egypt; ^4^Department of Biology, College of Science and Humanities, Prince Sattam bin Abdul Aziz University, Alkharj, Saudi Arabia; ^5^Department of Zoology, Faculty of Science, Suez Canal University, Ismailia, Egypt; ^6^Department of Biology, Faculty of Science, Albaha University, Alaqiq, Saudi Arabia; ^7^Department of Zoonoses, Faculty of Veterinary Medicine, Sohag University, Sohag, Egypt

**Keywords:** chronic toxoplasmosis, tamoxifen, histopathology, immunohistochemistry, real-time PCR

## Abstract

The global distribution of breast cancer and the opportunistic nature of the parasite have resulted in many patients with breast cancer becoming infected with toxoplasmosis. However, very limited information is available about the potential effects of tamoxifen on chronic toxoplasmosis and its contribution to the reactivation of the latent infection. The present study investigated the potential effects of tamoxifen on chronic toxoplasmosis in animal models (Swiss albino mice). Following induction of chronic toxoplasmosis and treatment with the drug for 14 and 28 days, the anti-parasitic effects of tamoxifen were evaluated by parasitological assessment and counting of *Toxoplasma* cysts. In addition, the effects of the drug on the parasite load were evaluated and quantitated using TaqMan real-time quantitative PCR followed by investigation of the major histopathological changes and immunohistochemical findings. Interestingly, tamoxifen increased the parasite burden on animals treated with the drug during 14 and 28 days as compared with the control group. The quantification of the DNA concentrations of *Toxoplasma P29* gene after the treatment with the drug revealed a higher parasite load in both treated groups vs. control groups. Furthermore, treatment with tamoxifen induced a series of histopathological and immunohistochemical changes in the kidney, liver, brain, and uterus, revealing the exacerbating effect of tamoxifen against chronic toxoplasmosis. These changes were represented by the presence of multiple *T. gondii tissue* cysts in the lumen of proximal convoluted tubules associated with complete necrosis in their lining epithelium of the kidney section. Meanwhile, liver tissue revealed multiple *T. gondii* tissue cysts in hepatic parenchyma which altered the structure of hepatocytes. Moreover, clusters of intracellular tachyzoites were observed in the lining epithelium of endometrium associated with severe endometrial necrosis and appeared as diffuse nuclear pyknosis combined with sever mononuclear cellular infiltration. Brain tissues experienced the presence of hemorrhages in pia mater and multiple *T. gondii* tissue cysts in brain tissue. The severity of the lesions was maximized by increasing the duration of treatment. Collectively, the study concluded novel findings in relation to the potential role of tamoxifen during chronic toxoplasmosis. These findings are very important for combating the disease, particularly in immunocompromised patients which could be life-threatening.

## Introduction

Toxoplasmosis is a parasitic zoonosis caused by a ubiquitous Apicomplexan protozoan named *Toxoplasma gondii* (Tenter et al., 2000). This parasite is distributed worldwide and about 30%–50% of the world population is found infected with the parasite (Tenter et al., 2000; Flegr et al., 2014). The definitive host of the parasite is *Felidae*, in which this obligate intracellular parasite replicates sexually in their intestine. A wide range of mammalian hosts, including humans and many other warm-blooded vertebrates, serve as intermediate hosts for the parasite (Dubey, 1996a). Humans contract infection by the parasite either horizontally by accidental ingestion of oocysts shed in feline feces or by ingestion of tissue cysts that persist in the muscles and nervous system of the intermediate hosts during consumption of undercooked meat or by consumption of food or drink contaminated with oocysts accidentally (Dubey, 2009). The parasite can also transmitted vertically by transplacental infection with tachyzoites passed from the mother to their offspring (Montaya and Liesenfeld, 2004; Montoya and Contopoulos-Ioannidis, 2021). This wide range of hosts and several modes of transmission reflects the importance of toxoplasmosis as a typical zoonotic disease for all warm-blooded animals, including humans (Tenter et al., 2000). Population structure studies of *T. gondii* have revealed that a few major clonal lineages predominate in different geographical regions of the globe. To date, four main genotypes of *T. gondii* strains have been recognized, namely I, II, III, and IV (Howe et al., 1997; Sanchez and Besteiro, 2021). Taken into account, Type I isolates or recombinants of types I and III are more likely to result in clinical toxoplasmosis but most isolates of *T. gondii* obtained from animals and genetically typed were type II or type III, irrespective of the clinical status of the animal (Howe and Sibley, 1995; Sanchez and Besteiro, 2021).

Regarding its life cycle, in the human host, orally ingested parasites are released from oocysts or tissue cysts (containing bradyzoite stages) and invade intestinal epithelial cells to transform into fast replicating tachyzoite stages. These later stages (tachyzoites) multiply intracellularly in a parasitophorous vacuole, which separates the parasite from the host cell cytoplasm (Sibley and Ajioka, 2008). In accordance with its clinical picture, the infection by the parasite is mostly either asymptomatic or mild picture represented by flu-like symptoms. However, the infection during pregnancy could be fatal to the fetus. Furthermore, Bradyzoite-containing brain cysts might become reactivated which results from reconversion into cytotoxic tachyzoites during secondary immune deficiency, causing toxoplasmic encephalitis (Joynson and Wreghitt, 2005).

The control of the diseases relies on two main strategies that include effective detective methods for diagnosis of the disease combined with developing novel drug targets for combating the infection, particularly in immunocompromised patients (Robert-Gangneux and Darde, 2012; Hassan et al., 2016). Taken into consideration, treatment of the disease in immunocompromised patients or pregnant women remains one of the major challenges for proper intervention for the disease. In these cases, the parasite remains dormant or less active inside tissues, making the treatment and complete elimination of the curative agent becomes a real challenge. Clearly, the development of novel drug targets for combating the disease in immunocompromised patients and understanding the mechanistic pathways and adverse effects of the available ones seem indispensable. Taken into consideration, as a result of the global distribution of breast cancer and the opportunistic nature of the parasite, many patients with breast cancer could be infected with toxoplasmosis (Molan and Rasheed, 2016; Kalantari et al., 2017; Wang et al., 2017). Several previous reports revealed the relatively high prevalence of toxoplasmosis in immunocompromised patients (Robert-Gangneux and Darde, 2012; Lu et al., 2015; Wang et al., 2017; Ali et al., 2019). The reactivation of the latent infection in immunocompromised patients could be life threatening (Carruthers and Suzuki, 2007; Schwartzman and Maguire, 2011; Sullivan and Jeffers, 2012; Pott and Castelo, 2013; Lu et al., 2015). Cancer patients are among the immunocompromised, and their immune status might result in reactivation of the latent infection by toxoplasmosis (Frenkel et al., 1978; Ali et al., 2019). It should be stressed that tamoxifen is widely known as a first-line adjuvant treatment of potent activity for primary prevention, treatment, and reduction of the risk of death due to different types of cancer in humans, particularly breast cancer (Gail et al., 1999; Lazzeroni et al., 2012; Nazarali and Narod, 2014). Tamoxifen is widely known as a selective estrogen-receptor modulator (SERM) belonging to the triphenylethylene group. Tamoxifen decreases the factors that enhance the growth of breast cells and increases the factors that decrease the growth of breast cells (Dowers et al., 2006; Hu et al., 2015). However, several serious side effects were reported with tamoxifen, including an increased risk of uterine cancer, vision troubles, stroke, pulmonary embolism, irregular periods, weight loss, and hot flashes, besides its harmful effects during pregnancy and breastfeeding (Jyoti et al., 2016). Revising the available literature, very limited previous studies investigated the potential adverse effects of tamoxifen on chronic toxoplasmosis and its influence on the reactivation of latent infection. In previous work, our research group performed a preliminary parasitological and serological study to investigate the effect of toxoplasmosis in experimentally infected animals (Selem et al., 2019). The same study concluded a significant increase in the parasite load for animals treated for 14 and 28 days together with a significant decrease in IgM titers and increase in IgG titers in treated animals as compared with control (Selem et al., 2019). However, the accompanied histopathological and immunohistochemical changes in different organs and quantification of the enhanced parasite burden using molecular techniques were not investigated. Given the above information, the present work aimed to better understandthe real contribution and influence of tamoxifen (TAM) on the reactivation of latent primary toxoplasmosis in Swiss Albino mice.

## Materials and Methods

### Ethical Considerations

The ethical approval of the present study was obtained from the guidance of the Research, Publication, and Ethics Committee of the National Research Center (NRC), Cairo, Egypt, which complies with all relevant Egyptian legislations in publication and research. The Institutional Review Board Number is 19139/2020.

### Materials

Tamoxifen (nolvadex) and sunflower oil were obtained from Sigma-Aldrich (Cairo, Egypt). All materials were of analytical grade.

### Parasite Material and Parasite Preparation

Tachyzoites of *T. gondii* (ME49 or Avirulent strain) were obtained from Zoonotic Diseases Department, National Research Center, Egypt. The step of preparation of parasite materials followed the protocol described elsewhere (Selem et al., 2019). Briefly, the parasite materials were maintained through serial intraperitoneal (i.p.) passage in mice. Tachyzoites were then collected from the peritoneal cavity of infected mice 72 h following the experimental infection. The parasites were counted by hemocytometer and then adjusted to 10^3^/ml by dilution of the organisms in the appropriate amount of saline followed by subcutaneous inoculation of each experimental animal (mice) with 1 ml solution.

### Animals and Experimental Protocol

**Figure 1:**
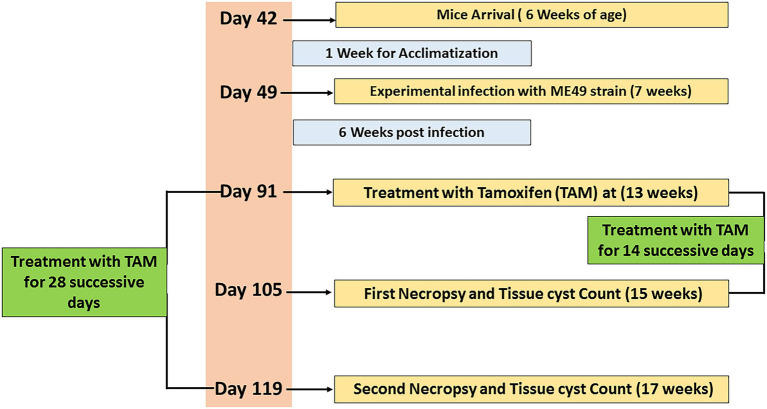
Timeline of the experimental protocol explaining the day (D) number, treatments, and sampling.

A total number of 64 laboratory-bred female Swiss albino mice (6 weeks old; weighing 20–25 g) were used in the present study. Animals were housed in clean well-ventilated cages under standard laboratory conditions (12-h light/dark cycle) and maintained in a suitable rearing environment in a temperature-controlled room (24°C–28°C). Animals were fed a standard diet with free access to food and water provided *ad libitum* throughout the experimental protocol. Furthermore, they underwent an acclimatization period (1 week) before the begining of the experiment. Animals were divided into four main groups (16 mice per group). The experimental protocol is shown in Figure 1 and the main groups were explained as follows:

- Group 1 (G1): negative control group; non-infected and non-treated mice.
- Group 2 (G2): positive control group (infected and non-treated) positive control mice.
- Group 3 (G3): infected and tamoxifen treated mice at dose of 10 mg/kg b.w daily (14 days treatment regimen).
- Group 4 (G4): infected and tamoxifen treated mice at dose of 10 mg/kg b.w daily (28 days treatment regimen).

At the age of 7 weeks, the infected non-treated and treated groups (G2, G3, and G4) were experimentally infected by the parasite using 0.1 ml of brain suspension contain 10^3^ cysts of previously infected mice by M49 strain (a virulent strain) by intraperitoneal injection for 6 weeks (Etewa et al., 2018). The infected-treated groups were also infected, as described in previously, then 6 weeks post-infection, these groups were treated with tamoxifen (nolvadex) by gavage at a dose of 10 mg/Kg body weight daily for 2 and 4 weeks (during the different treatment regimens) following the protocol described elsewhere (Perumal et al., 2005). Tamoxifen tablets were dissolved in sunflower oil and the dose was adjusted for each mouse according to its weight. At the end of each treatment time, infected and tamoxifen treated mice (G3 and G4) were sacrificed; their brains were dissected and examined for immediate direct parasitological assessment. In accordance with infected non treated groups (control positive group), half of the animals (*n* = 8) were sacrificed 14 days post treatment and served as control positive group for infected animals treated for 2 weeks. Meanwhile, the remaining animals (*n* = 8) of the control positive group were sacrificed 4 weeks post treatment and served as control infected non-treated group for those infected animals treated for 4 weeks. Mice feces were examined to exclude any parasitic infection as described elsewhere (Garcia, 1993).

### Parasitological Assessment

Following the treatment, mice were sacrificed 14 and 28 days post treatment. Brains were then removed and their suspensions were prepared in a tissue homogenizer (Wheaton, United States) using 1 ml saline. Later on, ~0.1 ml of each brain suspension was placed on a slide used for cyst enumeration and the cysts were counted in 10 high-power fields (HPF). The mean number of tissue cysts was determined for each animal followed by calculation of the mean numbers of cysts in each infected group as described elsewhere (Djurkoviae-Djakoviae and Milenkoviae, 2001).

### Histopathological Examination

Animals were treated with tamoxifen for 14 and 18 days then the both groups were sacrificed and tissue specimens from kidney, liver, uterus, and brain were gathered from the animals and fixed in 10% neutral buffer formalin (NBF) for histopathological examination. Tissue specimens were routinely processed, embedded in paraffin blocks, sectioned at 3–5 μm thickness and finally stained with Hematoxylin and Eosin (H&E) for allocation of the histopathological changes and detection of the parasite inside tissue. Furthermore, brain tissues, from the same groups, were processed to prepare tissue homogenate followed by preparation of smears stained with Giemsa for detection of the parasite (Bancroft and Gamble, 2008).

### Immunohistochemical Examination

In this step, tissue specimens from kidney were fixed in 10% NBF over-night then relocated into ethanol 70% to keep the antigenicity of *T. gondii*, routinely processed, embedded in paraffin, and sectioned at 3 μm thickness then mounted on positively charged slides. This step (IHC) followed the technique of streptavidin/biotin/peroxidase complex (ABC) using Peroxidase detection kit (Scy Tek Lab, United States), whereas the species specificity of the kit is anti-mouse and anti-rabbit. The retrieval of the antigen was performed using Proteinase K enzyme 0.1%. The examined samples were incubated with rabbit polyclonal anti *T. gondii* anti IgG (MP Biomedicals/Diagnostics Division/Orangeburg/United states). Tested sections were then counterstained with Mayer’s hematoxylin stain and examined under light microscopy (Haines and Clark, 1991).

### Ordinal Method for Validating Histopathologic and Immunohistochemical Scoring

Each animal was assigned a score based on tissue histopathological examination (Gibson-Corley et al., 2013). The samples were scored quantitatively and semiquantitatively, with assessment based on the visual field inspection of a minimum of 10 sections from each group. The histopathological lesions were then scored by an experienced veterinary pathologist who was unaware of the experimental treatments or their information in advance. Photographs were taken at a magnification of 40×, and the numbers of parasitic cysts were counted in 10 randomized areas (each 1 mm^2^; Atmaca et al., 2019; Ali et al., 2021). Furthermore, the histopathological lesions were scored according to set criteria: marked [massive] (41%–100% of tissue involved), moderate (21%–40% of tissue involved), mild (slight; 11%–20% of tissue involved), and minimal (0%–10% of tissue involved) by recording the nature and extent of the lesion and its frequency of occurrence in randomly selected sites in the tissue (Shackelford et al., 2002).

### Molecular Identification

In this step, brain tissue samples (20–25 mg) from animals were washed three times with sterile Phosphate-buffered saline (PBS; pH 7.4), then DNA was extracted from tissue samples of all groups using GF-1 Tissue DNA extraction kit (Cat.-No.GF-TD-050, Vivantis Co., Malaysia). The extraction steps were preceded as the manufacturer’s protocol. Real-Time Polymerase Chain Reaction (RT-PCR) was formed using ViPrime PLUS Taq qPCR Green Master Mix I (SYBR^®^ Green Dye, Cat QLMM12 Vivantis Co., Malaysia), according to the manufacture instruction. Briefly, 5 ng of DNA was mixed with 1X PCR master mix and 100 nmol of each sense and antisense primers (Table 1) in a total volume of 25 μl. The thermal cycler (MX30005P, Agilent) was programed as follow 95°C/5 min as initial denature and 40 cycles of 95°C/30 s and 60°C/1 min. The primers were designated using Laser gene DNA star software V15 and their sets are illustrated in Table 1. The dissociation curves were added after the end of the 40 cycles and data was adjusted to be collected by the end of annealing/extension cycle.

**Table 1:** Primer set used in the real-time polymerase chain reaction (RT-PCR).

P29 Q-f	CAGCATGGATAAGGCATCTG
P29 Q-r	GTTGCTCCTCTGTTAGTTCC

### Statistical Analysis

Data were analyzed using Statistical Program for Social Science (SPSS) version 15.0. The quantitative data were expressed as mean ± standard deviation (SD). Qualitative data were expressed as frequency and percentage. The statistical significance variance was considered significant when *p*-value was <0.05.

## Results

### Tamoxifen Increased the Parasitic Load

**Figure 2:**
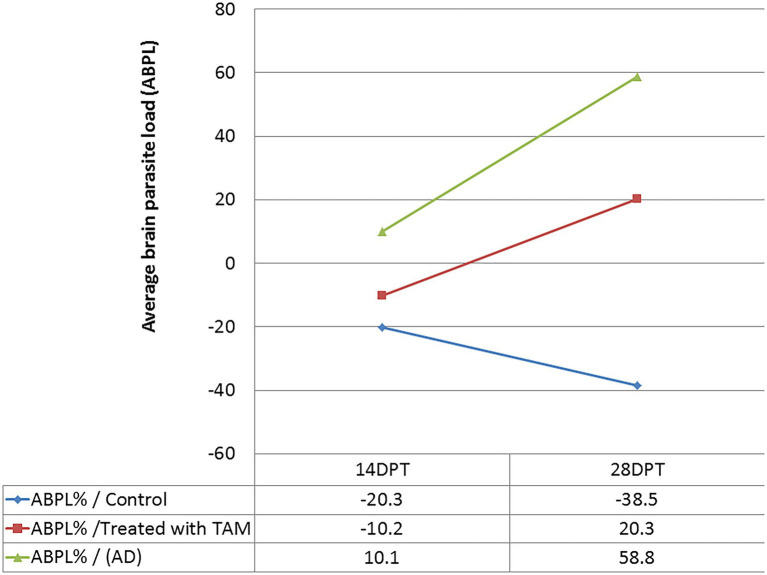
Average brain parasite load (ABPL) of tamoxifen-treated animals as compared with control at different time points.

The findings of parasite loads are shown in Figure 2 and Table 2. The results showed a significant (*p* = 0.0001–0.0045) increase in average brain parasitic load (ABPL) in the infected groups treated with tamoxifen (G3 and G4) for 14 and 28 days, by 10.1% and 58.8%, respectively.

**Table 2:** Average brain parasite load (ABPL) of tamoxifen-treated animals as compared with control at different time points.

DPI/DPT	Groups/average brain parasite load (ABPL/10 mg/brain)			
	N/BPL (Control infected mice)/(%)	N/BPL (infected mice treated with TAM)/(%)	ADG/(%)	Value of *p*
90 (IPL)	26.55			
14 DPT	21.15(−5.4)/(−20.3)	23.87(−2.7)/(−10.2)	+2.72 (10.1)	0.0045^*^
28 DPT	16.32(−10.2)/(−38.5)	31.92(+5.4)/(+20.3)	+15.6 (58.8)	0.0001^*^

ABPL, Average brain parasite load; IPL, Initial parasite load; DPT, Days post-treatment; DPI, Day post-infection; ADG, Average difference between treated and untreated groups.

* Means the statistical significance variance was considered significant when *p* < 0.05.

### Histopathological Findings

**Figure 3:**
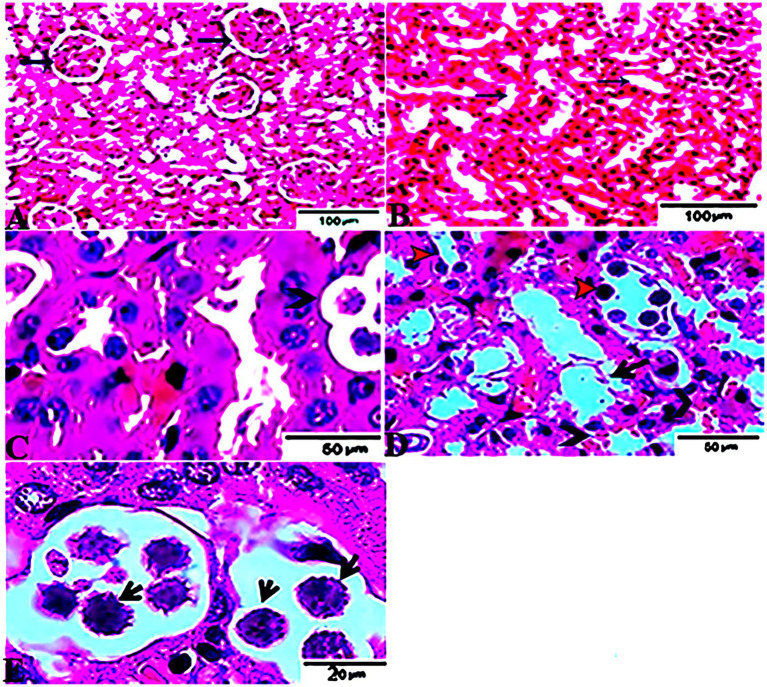
Photomicrograph of kidney sections from experimental mice groups stained with Hx&E stain: **(A,B)** represent tissue sections from control group showing normal histological architectures compromising in normal glomerular structure in the cortical area (**A**, arrows), surrounded with normal convoluted tubules. Normal tubular structures in renal medulla area (**B**, arrows); **(C)** renal tissue section from control positive group infected by *Toxoplasma gondii* showing; the presence of multiple *T. gondii* tissue cysts in the lumen of proximal convoluted tubules (arrow) associated with complete necrosis in their lining epithelium. **(D)** Kidney sections from the treated group for 14 days showing: the presence of marked infested multiple *T. gondii* tissue cysts in the lumen of proximal convoluted tubules (red arrowheads), severe necrosis in the lining epithelium of proximal convoluted tubules (arrows) associated with moderate extravasation of blood among renal tubules (arrowheads). **(E)** Renal tissue section from the treated group for 28 days showing: severe infested multiple *T. gondii* tissue cysts of ME49 Avirulent strain in the lumen of proximal convoluted tubules (arrows) associated with complete necrosis in their lining epithelium. The bar size of each picture was indicated under each picture.

**Figure 4:**
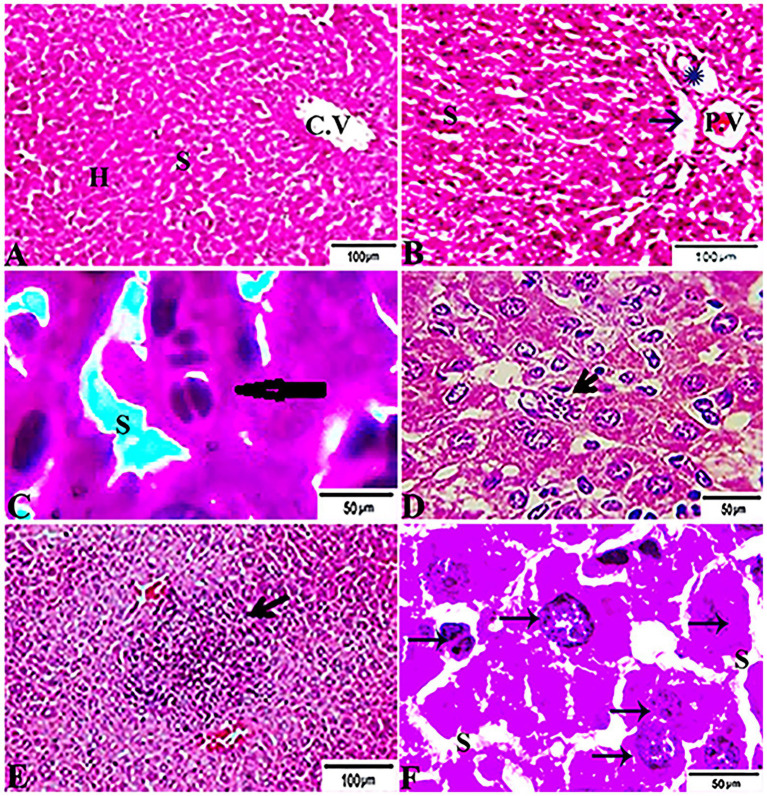
Photomicrograph of liver sections from experimental mice groups stained with H&E stain: **(A,B)** a mice liver from the control group demonstrating normal hepatic architectures. **(A)** Central vein (CV), sinusoids (S), and Hepatocytes (H). **(B)** Intact portal area; Portal Vein (P.V), Hepatic artery (arrow), and Bile duct (star). **(C)** Hepatic tissue section from the control positive group infected by *Toxoplasma gondii* showing; the presence of bradyzoites appeared as pseudocyst of *T. gondii* among degenerated hepatocytes (arrow). **(D,E)** Liver sections from the group treated for 14 days showing: **(D)** The presence of a cluster of tachyzoites appeared as pseudocyst of *T. gondii* among some degenerated hepatocytes (arrow), severe dilatation in hepatic sinusoids (S). **(E)** Focal aggregations of inflammatory cells in hepatic tissue (arrow). **(F)** Hepatic tissue section from treated group for 28 days showing: severe infested multiple *T. gondii* tissue cysts in hepatic parenchyma (arrows) which alters hepatic structure. The bar size of each picture was indicated under each picture.

**Figure 5:**
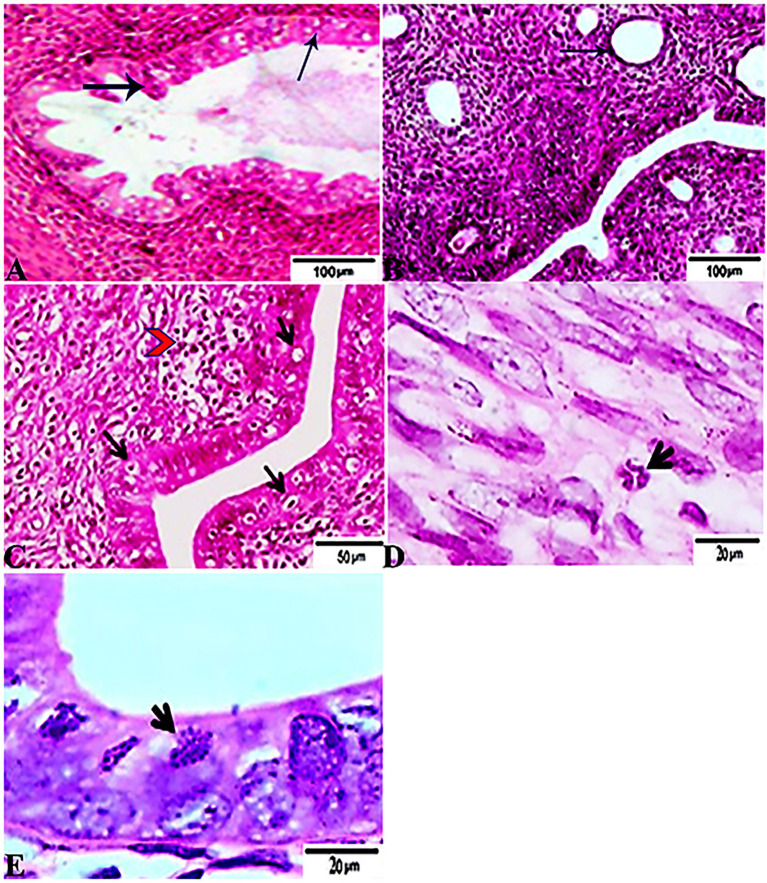
Photomicrograph of Uterus sections from experimental mice groups stained with Hx&E stain: **(A,B)** a mice uterus from the control group demonstrating normal uterine architectures. **(A)** Normal endometrial epithelium (arrows). **(B)** Normal endometrial glands (arrow). **(C)** Uterine tissue section from control positive group infected by *Toxoplasma gondii* showing; severe endometrial necrosis appeared as diffuse nuclear pyknosis in most of the endometrial epithelium (arrows), severe mononuclear cellular infiltration (red arrowheads). **(D)** Uterus sections from the treated group for 14 days showed: tachyzoites infestation in connective tissue stroma of the endometrial lamina propria submucosa (arrow). **(E)** Uterine tissue section from the treated group for 28 days showing: exhibited the presence of a cluster of tachyzoites intracellularly in the lining epithelium of endometrium (arrow). The bar size of each picture was indicated under each picture.

**Figure 6:**
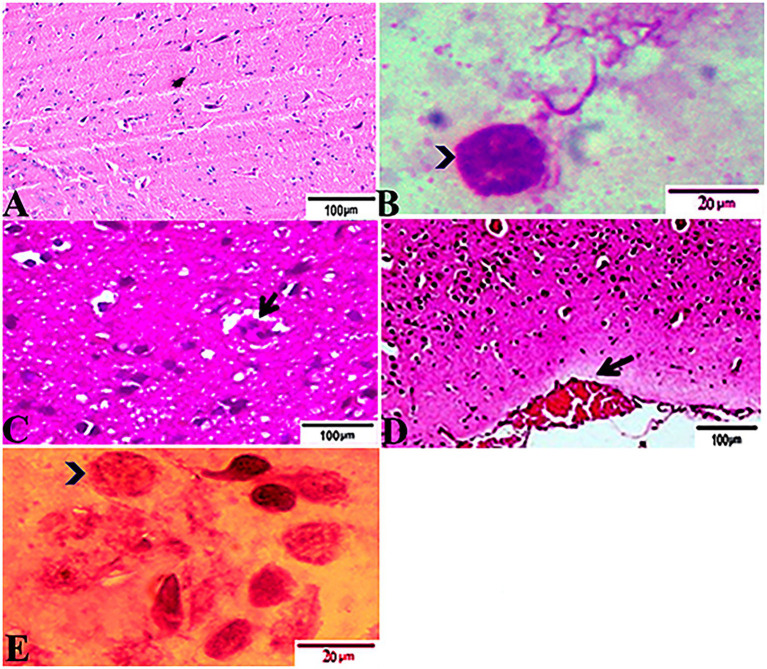
Photomicrograph of brain sections from experimental mice groups stained with Hx&E stain: **(A)** a mice brain from the control group demonstrating normal brain architectures. **(B)** Brain tissue section from control positive group infected by *Toxoplasma gondii* showing; the presence of *T. gondii* cysts (arrowhead). **(C)** Brain sections from the group treated for 14 days showing: degenerated *T. gondii* tissue cysts in the white matter of the infected cerebrum (arrow). **(D,E)** Brain tissue section from the treated group for 28 days showing: **(D)** hemorrhages in pia mater (arrow), **(E)** exhibited the presence of multiple *T. gondii* tissue cysts in brain tissue (arrowhead). The bar size of each picture was indicated under each picture.

The histopathological features of the tissue specimens from the kidney, liver, uterus, and brain are shown in Figures 3–6. As depicted, Figures 3A,B reveal normal histological architectures of kidney for control negative healthy group (G1), compromising a normal glomerular structure in the cortical area surrounded by normal convoluted tubules and normal tubular structures in the renal medulla area. Meanwhile, Figure 3C reveals the renal tissue from the control positive group (G2) infected by *T. gondii* showing the presence of multiple *T. gondii* tissue cysts in the lumen of proximal convoluted tubules associated with complete necrosis in their lining epithelium. Regarding the kidney sections from the group treated with tamoxifen for 14 days (G3), they showed the presence of marked infested multiple *T. gondii* tissue cysts in the lumen of proximal convoluted tubules, severe necrosis in the lining epithelium of proximal convoluted tubules associated with moderate extravasation of blood among renal tubules. On the other hand, the histopathological sections from renal tissue in the infected group (G4) treated with tamoxifen for 28 days showed severely infested multiple *T. gondii* tissue cysts of ME49 Avirulent strain in the lumen of proximal convoluted tubules (associated with complete necrosis in their lining epithelium; Figure 3E). The histopathological features in the liver of experimental groups are illustrated in Figure 4. As shown, Figures 4A,B depict the liver section from the control group (G1) demonstrating normal and intact hepatic architectures including central vein, sinusoids, hepatocytes, portal area, portal vein, and bile duct. Meanwhile, the hepatic tissue sections from the control positive group (G2) infected by *T. gondii* showed the presence of bradyzoites appearing as pseudocysts of *T. gondii* among degenerated hepatocytes (Figure 4C). In accordance with the group treated for 14 days (G3), which is shown in Figures 4D,E, liver sections revealed the presence of a cluster of tachyzoites which were represented by the presence of pseudocysts of *T. gondii* among some of the degenerated hepatocytes, sever dilatation in hepatic sinusoids, and focal aggregations of inflammatory cells in hepatic tissue (arrow). On the other hand, the hepatic tissue section from the infected group treated for 28 days (G4) experienced severe infested multiple *T. gondii* tissue cysts in hepatic parenchyma which altered the structure of hepatocytes. As illustrated, Figures 5A,B reveal the normal uterine architectures of the uterus sections of the control groups (G1). Meanwhile, the uterine tissue section from the control positive group (G2) infected by *T. gondii* showed severe endometrial necrosis appeared as diffuse nuclear pyknosis in most of the endometrial epithelium combined with severe mononuclear cellular infiltration (Figure 5C). On the other hand, uterus sections from the infected group treated for 14 days (G3) exhibited tachyzoites infestation in connective tissue stroma of the endometrial lamina propria submucosa (Figure 5D). However, the presence of a cluster of tachyzoites intracellularly in the lining epithelium of endometrium was the main characteristic of uterine tissue sections from the infectedreated group treated for 28 days (G3; Figure 5E). The normal brain architectures from the control healthy group (G1) are shown in Figure 6A, while Figure 6B exhibits the brain tissue section from the control positive group (G2) infected by *T. gondii* showing the presence of *T. gondii* cysts. Regarding the group treated for 14 days (G3), brain sections from this group showed degenerated *T. gondii* tissue cysts in the white matter of the infected cerebrum (Figure 6C). On the other hand, brain from the group treated for 28 days (G4) showed hemorrhages in the pia mater and revealed the presence of multiple *T. gondii* tissue cysts in brain tissue (Figures 6D,E).

### Immunohistochemically Findings

**Figure 7:**
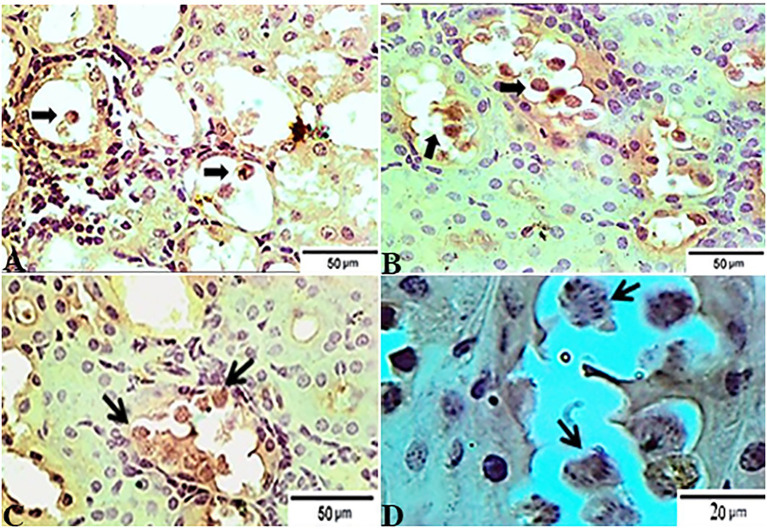
Photomicrograph of immunohistochemical staining for anti-*Toxoplasma gondii* anti IgG in kidney sections from experimental mice groups showed positive golden brown immunoreactive *T. gondii* tissue cysts fill most of the infected renal tubules using IHC technique, DAB stain; **(A)** control positive group infected with ME49 Avirulent strain of *T. gondii* showing mild tissue IHC reaction. **(B)** Treated group for 14 days showed moderate IHC tissue reaction. (**C** magnified in **D**): from the treated group for 28 days; strong positive results by IHC represented by the presence of multiple golden-brown positive immunoreactivity *T. gondii* tissue cysts nearly occludes the infected renal tubules. The bar size of each picture was indicated under each picture.

In accordance with the immunohistochemical findings, which are shown in Figure 7, kidney sections from experimental mice groups showed positive golden brown immunoreactive *T. gondii* tissue cysts filling most of the infected renal tubules using the IHC technique, DAB stain. Figure 7A depicts the control positive group (G2) infected with *T. gondii* experiencing mild tissue IHC reaction. Meanwhile, the treated group for 14 days (G3) showed moderate IHC tissue reaction (Figure 7B) and those infected groups treated with tamoxifen (G4) for 28 days experienced strong positive results by IHC represented by the presence of multiple golden-brown positive immunoreactivity *T. gondii* tissue cysts nearly occludes the infected renal tubules (Figure 7).

### Histopathologic and Immunohistochemical Scoring

**Figure 8:**
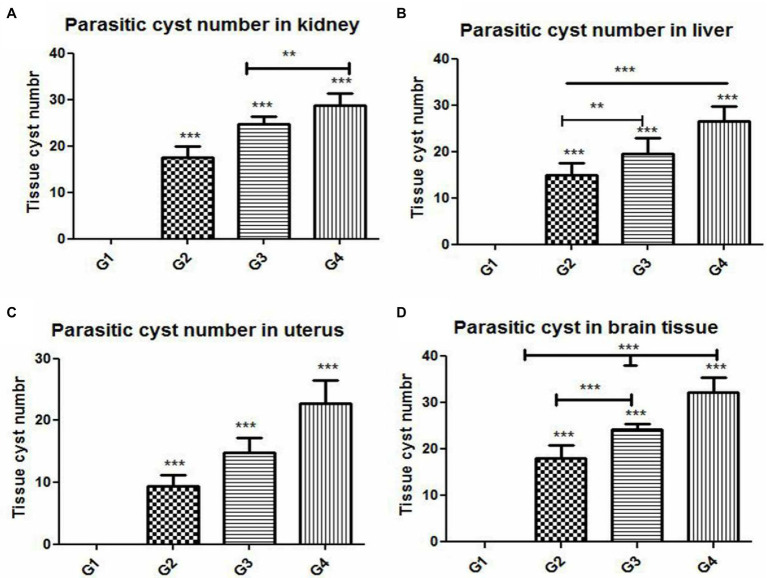
Histomorphometry graph showing quantitative measurements of parasitic cyst in **(A)** kidney, **(B)** liver, **(C)** uterus, and **(D)** brain tissue among the experimental groups. Data are expressed as means ± standard deviations. Significant differences vs. the control group are marked by different asterisks through one-way ANOVA with Tukey’s *post-hoc* test: ^**^p ≤ 0.01, ^***^p ≤ 0.001.

The results of histopathologic and immunohistochemical scoring for different organs following treatment of infected animals with tamoxifen for 14 and 28 days vs. the control group are shown in Table 3 and Figure 8.

**Table 3:** Lesions scoring for reported pathological lesions of various organs in the experimental groups.

Organ	Lesion	G1	G2	G3	G4
Kidney	Renal tubular necrosis	Normal	Mild	Moderate	Sever
	Interstitial hemorrhage	Normal	Minimal	Mild	Sever
	Congestion in vasculature	Normal	Moderate	Sever	Sever
	Parasitic cyst infestation	Normal	Mild	Moderate	Sever
	Intensity of immunohistochemistry marker (rabbit polyclonal anti *Toxoplasma gondii* anti IgG)	–	Mild	Moderate	Sever
Liver	Hepatocellular degeneration	Normal	Moderate	Sever	Sever
	Sinusoidal dilatation	Normal	Moderate	Moderate	Sever
	Focal inflammatory cellular aggregation	Normal	Minimal	Moderate	Sever
	Parasitic cyst infestation	Normal	Mild	Moderate	Sever
Uterus	Endometrial necrosis	Normal	Moderate	Sever	Sever
	Inflammatory cellular infiltration	Normal	Moderate	Sever	Sever
	Parasitic cyst infestation	Normal	Mild	Moderate	Sever
Brain	Hemorrhages	Normal	Mild	Mild	Moderate
	Degenerated nerve cells	Normal	Mild	Moderate	Sever
	Parasitic cyst infestation	Normal	Mild	Moderate	Sever

### Molecular Results

In this step, the DNA concentrations of *Toxoplasma P29* gene were quantified after the treatment with tamoxifen in presence of reference samples (untreated; G2). The tested samples of those infected groups treated with tamoxifen (G3 and G4) revealed marked variation in the product quantities. In this respect, the CT values and absolute quantity of both the standard and the treated samples are shown in Table 4. Meanwhile, Table 5 illustrates the variable change in the *T.gondii* load after treatment of infected animals with the tamoxifen in comparison with the infected, non-treated animals (G2). Importantly, the present study revealed that the treatment with tamoxifen (G3 and G4) increased the parasite load in both treatment regimens (14 and 28 days post-treatment).

**Table 4:** The CT values and absolute quantity of both the standard and the treated samples.

Experiment	Group	Group type	Ct (dRn)	Quantity (copies)
NTC	G1	NTC (non-infected- non treated)	No Ct	No Ct
Positive control	G2-a	Standard infected non treated (14 DPT)	39.07	4.80E-06
	G2-b	Standard infected non treated (14 DPT)	23.48	4.80E-02
	G2-c	Standard infected non treated (14 DPT)	32.17	4.80E-03
	G2-d	Standard infected non treated (14 DPT)	33.09	4.80E-02
	G2-e	Standard infected non treated (28 DPT)	24.97	4.80E-01
	G2-f	Standard infected non treated (28 DPT)	31.7	4.80E-04
	G2-g	Standard infected non treated (28 DPT)	32.6	4.80E-03
	G2-h	Standard infected non treated (28 DPT)	31.13	4.80E-03
Treated	G3-a	Infected treated with TAM (14 DPT)	20.52	2.10E-01
	G3-b	Infected treated with TAM (14 DPT)	20.22	3.08E-01
	G3-c	Infected treated with TAM (14 DPT)	22.37	3.08E-02
	G3-d	Infected treated with TAM (14 DPT)	25.21	3.08E-01
	G4-a	Infected treated with TAM (28 DPT)	20.09	2.68E-01
	G4-b	Infected treated with TAM (28 DPT)	20.24	2.46E-03
	G4-c	Infected treated with TAM (28 DPT)	21.15	2.46E-02
	G4-d	Infected treated with TAM (28 DPT)	25.76	2.46E-01

**Table 5:** The variable change in the *Toxoplasma gondii* load after treatment with the anticancer in comparison with the infected, non-treated animals.

Sample ID	Quantity
C- (Infected untreated) sample	0.00E+00
S1 (Treated for 14 DPT)	3.96E-02
S2 (Treated for 14 DPT)	4.01E-01
S3(Treated for 14 DPT)	3.26E-02
S4 (Treated for 14 DPT)	5.71E-03
S5 (Treated for 28 DPT)	9.80E-02
S6 (Treated for 28 DPT)	9.82E-01
S7 (Treated for 28 DPT)	9.55E-02
S8 (Treated for 28 DPT)	9.90E-03

## Discussion

Toxoplasmosis is considered one of the major zoonotic parasites of worldwide distribution (Tenter et al., 2000; Flegr et al., 2014). Despite several *Toxoplasma* studies in the last few decades, many questions are still unanswered, particularly those related to the clinical course and prognosis of the disease in immunocompromised patients, i.e., cancer and AIDS patients. Reactivation of latent infection and the potential effects of using several anticancer drugs in cancer patients co-infected with toxoplasmosis remain an additional challenge. The present study reports interesting parasitological and molecular findings about the potential association of tamoxifenwith the activation of latent infection during chronic toxoplasmosis. The findings were supported by a series of histopathological and immunohistochemical examinations. As mentioned above, tamoxifen is widely known as a potent anticancer agent has been used in the treatment of the early stages of breast cancer, particularly in women with marked ovarian estrogenic activity (Clarke et al., 2003; Criscitiello et al., 2010). Given its immunomodulatory effects, tamoxifen safely reduced the recurrence of breast cancer and risks of death over these last decades (Behjati and Frank, 2009; Early Breast Cancer Trialists’ Collaborative Group, 2011). Furthermore, tamoxifen exhibited a wide range of activity against several parasites including *Plasmodium falciparum*, *Taenia solium*, *Leishmania amazonensis*, *Trypanosoma cruzi*, and *T. gondii*, among others (Miguel et al., 2008; Escobedo et al., 2013; Dittmar et al., 2016; Landoni et al., 2019; Weinstock et al., 2019). However, very limited information is available about the possible link between tamoxifen and chronic toxoplasmosis (Selem et al., 2019).

It is noteworthy to mention that the central nervous system (CNS) and muscle had the highest frequency of parasites during chronic infection (Bierly et al., 2008; Gulinello et al., 2010; Parlog et al., 2015; Mose et al., 2017; Etewa et al., 2018; Zhao and Ewald, 2020). Furthermore, as per parasite biology, the formation of tissue cysts might occur in various tissues other than brain and muscle, including lungs, heart, liver, spleen, kidneys, and uterus, among others (Dubey, 1997; Renoult et al., 1997; Dubey et al., 1998; Haziroğlu et al., 2003; Galvan-Ramirez et al., 2018; Matta et al., 2021). In these cases, the parasite can persist for the life of the intermediate host but they are not considered immune-privileged (Renoult et al., 1997; Zhao and Ewald, 2020; Matta et al., 2021). In the present work, as shown in Table 2; Figure 2, the parasitological examination revealed a significant increase in the average brain parasitic load in infected animals treated with tamoxifen for 14 and 28 days (G3 and G4) as compared to the control positive group (G2). In a previous work (Selem et al., 2019), we reported a significant increase in parasitic burden in animals treated with tamoxifen for 14 and 28 days. In the same work, there was observed a significant increase in IgM and IgG titers of the parasite in groups treated with tamoxifen for 4 weeks, which is consistent with the present findings. Similarly but in other parasites, a previous study reported that tamoxifen increased parasite load during the infection by *Plasmodium berghei* and exacerbated the symptoms of infected animals combined with suppression of the immune response (Cervantes-Candelas et al., 2021). The possible explanation for this effect could be attributed to the immunomodulatory role of tamoxifen and the potential involvement of different cellular compartments of the immune system which was reported in other parasites (Cervantes-Candelas et al., 2021).

To the knowledge of the authors, histopathological examination and immunohistochemistry represent important diagnostic tools for exploring the distribution of the different stages of the parasite in the infected tissues and its antigenic residues (Uggla et al., 1987). Interestingly, the present study revealed the dissemination and clustering of *T. gondii* tachyzoites in several organs of infected animals in those groups treated with tamoxifen for 14 and 28 days (G3 and G4). In this respect, localization of tissue cysts mainly occurred in the kidney and brain of experimental animals. Furthermore, there was a marked change in the histological structures of the examined organs of the infected and treated groups, particularly this group infected and treated with tamoxifen for 28 days (G4), as compared with the control positive group (G2). The reported histopathological changes included the presence of *T. gondii* tissue cysts in those organs, diffuse nuclear pyknosis (uterus), inflammatory cells infiltration, moderate hemorrhages in pia mater (brain), and severe necrosis in the lining epithelium of proximal convoluted tubules (kidney) which was associated with moderate extravasation of blood among renal tubules besides moderate focal aggregations of inflammatory cells in hepatic tissue and complete necrosis in some organs. These findings are in accordance with a previous study (Egan et al., 2005) revealed that *T. gondii* infection is characterized by a wide spreading of tachyzoites which can invade any cell type. Meanwhile, the chronic infection stage is accompanied by the differentiation of tachyzoites into bradyzoites that form tissue cysts within the brain and skeletal muscle. In the same line, a previous study (Sheng-Wei et al., 2011) reported a series of histopathological changes in mice infected with *T. gondii* Prugniaud strain that included degeneration of affected tissues, necrosis, and infiltration of inflammatory cells in several organs that were detected in the early period of *T. gondii* tachyzoites infection in mice. This was followed by the co-habit phenomenon of non-specific infection with cysts in the brain. The present results are also in harmony with several previous studies that concluded that gliosis, inflammatory cells infiltration, the presence of single or multiple tissue cysts, and rupture of tissue cyst are the most common histopathological characteristics of the chronic toxoplasmosis (Dubey, 1996b; Gulinello et al., 2010; Parlog et al., 2015; Dubey et al., 2016; Fuentes-Castro et al., 2017). The possible explanation of these findings is the fact that toxoplasmosis induces a marked inflammatory damage which is usually associated with enhancement of the pro-inflammatory cytokines and recruitment of multiple inflammatory cells which in turn targets the parasite replication and might be fatal to the host (Kelly et al., 2005; Mukhopadhyay et al., 2020; Sanchez and Besteiro, 2021).

Similarly, the IHC findings revealed a strong positivity to the tissue cysts from kidney of infected groups treated with tamoxifen (G3 and G4) vs. the control group (G2). Our present findings are consistent with several previous reports that revealed a series of immunohistochemical findings which included the presence of *Toxoplasma* antigen combined with mild-to-moderate congestion, focal or multifocal mon and polymorphonuclear inflammatory cell infiltrate (Silva et al., 2013; Hanafiah et al., 2017). Taken into account, toxoplasmic IgG titer reported to appear 2 weeks following the infection and reached its peak after 3 months. Later on, the Toxoplasmic IgG titer remains at a plateau level for 6 months then the IgG level decreased slowly after 1 year to lower levels. It seems that the antigen-binding avidity of IgG antibody slowly increases during the first 4 months following the infection, and therefore, IgG could be more useful and of potential diagnostic value during chronic stage of toxoplasmosis (Villard et al., 2016).

It should be stressed that the molecular methods, including several polymerase chain reaction (PCR)–based techniques, are among the most accurate methods for the detection of the parasite in biological samples (Zhao et al., 2010; Gutierrez et al., 2012; El-Settawy et al., 2016; Marino et al., 2017). Among others, real-time PCR is a highly sensitive and specific laboratory technique used for detection of toxoplasmosis and quantification of the parasite load and its genotypes (Lin et al., 2000). The present work also evaluated the effects of tamoxifen on parasite growth and replication using real-time PCR. Importantly, p29 (GRA7) gene is a protein present in the dense granules of *T. gondii* (Bonhomme et al., 1998). This protein is secreted by bradyzoites and showed a potential prognostic value for determining the stage of the disease (Cesbron-Delauw, 1994; Bonhomme et al., 1998; Dunn et al., 2008). In our present work, the DNA concentrations of *Toxoplasma P29* gene have been quantified after treatment of infected animals with tamoxifen (G3 and G4). As shown, all tested samples gave positive results with clear variation in the product quantities as compared with control samples. The present findings are in harmony with those reported elsewhere (Chen et al., 2019). This effect could be driven by the immunomodulatory effect of tamoxifen which was also reported in a other parasites (Cervantes-Candelas et al., 2021). Given the above information, the molecular results confirmed the findings obtained by parasitological, histopathological, immunohistochemical methods. Collectively, the present study pointed out the potential benefits of the combined use of parasitological, molecular, histopathological, and immunohistochemical methods for exploring the potential effects of drug targets adopted for combating the disease (Meixner et al., 2020).

## Conclusion

The present work concluded that treatment of chronically infected mice with *T. gondii* with tamoxifen has resulted in exacerbation of infection, and therefore could be associated with enhancement of the infection and possible reactivation of latent infection. These findings were confirmed through a series of parasitological, molecular, histopathological, and immunohistochemical methods. Clearly, our study suggests that screening of toxoplasmosis in immunocompromised individuals, particularly those with breast cancer or those on adjuvant therapy with tamoxifen, before and during the course of treatment could be helpful for earlier detection of any upcoming reactivation of latent infection. It would be interesting to investigate the different immunological mechanisms underlying these effects combined with the determination of the parasite load on muscle tissue following treatment with tamoxifen. Further future similar studies on tamoxifen and toxoplasmosis in humans and other mice strains are suggested.

## Data Availability Statement

The original contributions presented in the study are included in the article/supplementary material, further inquiries can be directed to the corresponding authors.

## Ethics Statement

The animal study was reviewed and approved by the Research, Publication, and Ethics Committee of the National Research Center (NRC), Cairo, Egypt, which complies with all relevant Egyptian legislations in publication and research. The institutional Review Board Number is 19139/2020.

## Author Contributions

AB, HAME, RS, AM, KAA, EH, AA, and EE involved in the conception of the idea, methodology design, performed data analysis and interpretation, and prepared the manuscript for publication and revision. All authors contributed to the article and approved the submitted version.

## Funding

This work was financially supported by project number 12010135, which received funding through a grant from National Research Centre (NRC), Egypt, 2019–2022.

## Conflict of Interest

The authors declare that the research was conducted in the absence of any commercial or financial relationships that could be construed as a potential conflict of interest.

## Publisher’s Note

All claims expressed in this article are solely those of the authors and do not necessarily represent those of their affiliated organizations, or those of the publisher, the editors and the reviewers. Any product that may be evaluated in this article, or claim that may be made by its manufacturer, is not guaranteed or endorsed by the publisher.
